# Evaluating mortality and recovery of extreme hyperbilirubinemia in critically ill patients by phasing the peak bilirubin level: A retrospective cohort study

**DOI:** 10.1371/journal.pone.0255230

**Published:** 2021-08-05

**Authors:** Hong Seok Han, Chi-Min Park, Dae-Sang Lee, Dong Hyun Sinn, Eunmi Gil

**Affiliations:** 1 Department of Surgery, Samsung Medical Center, Sungkyunkwan University School of Medicine, Seoul, Republic of Korea; 2 Department of Critical Care Medicine, Samsung Medical Center, Sungkyunkwan University School of Medicine, Seoul, Republic of Korea; 3 Department of Trauma Surgery, Ujeongbu St. Mary’s Hospital, College of Medicine, The Catholic University of Korea, Seoul, Republic of Korea; 4 Division of Gastroenterology, Department of Internal Medicine, Samsung Medical Center, Sungkyunkwan University School of Medicine, Seoul, Republic of Korea; Heidelberg University Hospital, GERMANY

## Abstract

**Background:**

Hyperbilirubinemia is a devastating complication in patients admitted to an intensive care unit (ICU). The sequential organ failure assessment (SOFA) score classifies hyperbilirubinemia without further detailed analyses for bilirubin increase above 12 mg/dL. We evaluated whether the level of bilirubin increase in patients with extreme hyperbilirubinemia (total bilirubin ≥ 12 mg/dL) affects and also helps estimate mortality or recovery.

**Methods:**

A retrospective cohort analysis comprising 427 patients with extreme hyperbilirubinemia admitted to the ICU of Samsung Medical Center, Seoul, Korea between 2011 and 2015 was conducted. Extreme hyperbilirubinemia was classified into four grades: grade 1 (12–14.9 mg/dL), grade 2 (15–19.9 mg/dL), grade 3 (20–29.9 mg/dL), and grade 4 (≥ 30 mg/dL). These grades were then assessed for their association with hospital mortality and recovery from hyperbilirubinemia to SOFA grade (point) 2 or below (total bilirubin < 6 mg/dL). The influences of various factors, some of which caused extreme hyperbilirubinemia, while others induced bilirubin recovery, were assessed.

**Results:**

A total of 427 patients (mean age: 59.8 years, male: 67.0%) were evaluated, and the hospital mortality for these patients was very high (76.1%). Extreme hyperbilirubinemia was observed in 111 (grade 1, 26.0%), 99 (grade 2, 23.2%), 131 (grade3, 30.7%), and 86 (grade 4, 20.1%) patients with mortality rates of 62.2%, 71.7%, 81.7%, and 90.7%, respectively (p < 0.001). The peak bilirubin value correlated with the mortality (odds ratio [OR], 1.09; 95% confidence interval [CI], 1.04–1.15, p < 0.001). Compared to those with grade 1 extreme hyperbilirubinemia, the mortality rate gradually increased as the grade increased (OR [95% CI]: 1.92 [0.70–5.28], 3.55 [1.33–9.48], and 12.47 [3.07–50.59] for grades 2, 3 and 4, respectively). The main causes of extreme hyperbilirubinemia were infection including sepsis and hypoxic hepatitis. The recovery from hyperbilirubinemia was observed in 110 (25.8%) patients. Mortality was lower for those who recovered from hyperbilirubinemia than for those who did not (29.1% vs. 92.4%, p < 0.001). The favorable factors of bilirubin recovery were albumin and ursodeoxycholic acid (UDCA).

**Conclusions:**

This study determined that the level of extreme hyperbilirubinemia is an important prognostic factor in critically ill patients. We expect the results of this study to help predict the clinical course of and determine the optimal treatment for extreme hyperbilirubinemia.

## Introduction

Multiple organ failure is one of the most common causes of morbidity and mortality in an intensive care unit (ICU) [1, 2]. Hepatic dysfunction is considered the most important prognostic factor in such patients [1–5]. It is well-established that a high level of serum bilirubin not only indicates liver dysfunction but also contributes to an imbalanced immune response, aggravated bacterial infections, and impaired renal or cardiac function; furthermore, the increased bilirubin levels are closely associated with increased mortality [3–7]. The sequential organ failure assessment (SOFA) score grades liver dysfunction in the range 0–4 at respective bilirubin cut-off bilirubin levels of 1.2, 2.0, 6.0, and 12.0 mg/dL. However, there is no further grading scheme for bilirubin exceeding 12.0 mg/dL. In clinical practice, some patients suffer from extreme hyperbilirubinemia (≥ 12.0 mg/dL); this condition is poorly understood, results from multiple possible causes, and leads to diverse clinical outcomes [8].

Organ failure is not an all-or-nothing phenomenon, but rather a continuum of alteration, and liver failure is not an exception [1, 2, 9]. Although extreme hyperbilirubinemia is not subdivided further in the SOFA scoring system, those suffering from it might be a heterogeneous population exhibiting distinct survival rates. There have been no large-scale studies to date showing whether the analysis of mortality in critically ill patients with extreme hyperbilirubinemia admitted to the ICU can be useful. In this study, we assessed the hospital mortality and recovery according to subdivided extreme hyperbilirubinemia level and also evaluated the factors causing extreme hyperbilirubinemia. In addition, this study analyzes the relationship between these factors and recovery from extreme hyperbilirubinemia.

## Methods

### Study design and patients

This is a retrospective cohort study involving patients who were admitted to the ICU of Samsung Medical Center, Seoul, South Korea in 2011–2015. We included all critically ill patients admitted to the ICU who developed a serum total bilirubin greater than or equal to 12 mg/dL at least once during their ICU stay. Among these patients, we excluded those who met the following exclusion criteria: 1) age under 19 years, 2) patients who underwent or had received liver transplantation, and 3) patients whose serum total bilirubin already exceeded 12 mg/dL upon ICU admission. Ultimately, 427 patients were included in this study (S1 Fig). This study protocol was reviewed and approved by the Institutional Review Board at Samsung Medical Center (IRB No. 2016-03-058). Because the study was a retrospective analysis of existing administrative and clinical data, the requirement to obtain informed patient consent was waived by the Institutional Review Board.

### Definition and classification of hyperbilirubinemia

All serum bilirubin levels during patients’ ICU stay were collected. Serum bilirubin levels were measured by colorimetric assay (diazonium salt/ion with blank) methods from the lowest level of detection to highest level of detection using Roche Modular DP equipment and the Bilirubin Total Gen. 3 reagent (Roche Diagnostics Corporation). Based on the peak serum bilirubin level, patients were classified into one of four further scales; grade 1 (≥ 12 mg/dL and < 15 mg/dL), grade 2 (≥ 15 mg/dL and < 20 mg/dL), grade 3 (≥ 20 mg/dL and < 30 mg/dL), and grade 4 (≥ 30 mg/dL). Furthermore, we investigated laboratory analyses related to liver dysfunction as follows: aspartate aminotransferase (AST), alanine aminotransferase (ALT), alkaline phosphatase (ALP), γ(gamma)-glutamyl transferase (GGT), international normalized ratio (INR), albumin, c-reactive protein (CRP), and lactate [10–13]. The laboratory results above for each patient were statistically analyzed as the mean values obtained during the 15 days before and after the first incidence of serum total bilirubin greater than 12 mg/dL after ICU admission. Then, the median values (IQRs) were calculated for the total patient population or specific groups (e.g., survivors or recovery group).

We evaluated the major causes of extreme hyperbilirubinemia. Various factors contributing to total bilirubin increases in extreme hyperbilirubinemia were identified, and almost all patients had multi-factorial causes rather than a single factor. It was very difficult to select a single, primary cause, so we selected the two most important factors in order of influence based on medical records, clinical features, and findings regarding the pattern and timing of bilirubin increase. These major causes were categorized as obstructive (stone, neoplasm, and stricture) and non-obstructive (hypoxic hepatitis, infection including sepsis, primary liver disease, traumatic or surgical liver injury, pigment overload, drug, other organ failure–related jaundice, and long-term nil-per-os [NPO]). Obstructive hyperbilirubinemia is defined as hyperbilirubinemia caused by biliary obstructions due to biliary stone, neoplasm, or duct stricture of biliary system identified by ultra-sonography (US) and abdominal computerized-tomography (CT). We categorized non-obstructive hyperbilirubinemia as that occurring in the absence of obstructive lesions. Pigment overload refers to hemolysis, massive bleeding and/or transfusion, hematoma resorption, disseminated intravascular coagulation (DIC), hemolytic uremic syndrome, and hematologic disorder related hemolysis [8, 14]. The drug category included any drugs or toxic materials causing liver damage, such as chemotherapy agents, antibiotics, total parenteral nutrition (TPN), herbal medicine, etc. [8, 10, 11].

Direct and indirect hyperbilirubinemia were investigated using total and direct bilirubin. The reference ratio for direct hyperbilirubinemia has not been fully established, but several definitions have been proposed: direct bilirubin (DB) / total bilirubin (TB) ≥ 50% or 60% or 70% [15–17]. We collected data according to these three definitions and chose the 60% value for definition and analysis.

### Data collection

The following clinical data were retrospectively obtained from the ICU database and chart review: age, sex, body mass index (BMI), history of alcohol drinking and smoking, location before ICU admission, reasons for ICU admission, surgical status at the time of ICU admission, and comorbidities including diabetes mellitus (DM), hypertension (HTN), dyslipidemia, chronic liver disease, hepatobiliary-pancreatic (HBP) cancer, solid cancer (excluding HBP cancer), and hematologic cancer. HBP cancer refers to not only primary cancer but also metastatic cancer in the liver, bile duct, and pancreas. Chronic liver disease refers to liver cirrhosis, chronic viral hepatitis including hepatitis B and hepatitis C, alcoholic liver disease, autoimmune hepatitis, and drug-induced and toxic hepatitis. We also investigated congenital bilirubin metabolism disorders and identified 5 patients with Gilbert’s syndrome. However, patients with this syndrome had long been treated before ICU admission and the disorder was not related to extreme hyperbilirubinemia.

We assessed the presence of sepsis and shock (septic, cardiogenic, and hypovolemic) at ICU admission. A modified SOFA score (not including Glasgow Coma Scale) was applied at ICU admission [18].

We investigated clinically important factors that might have influenced the development of, or recovery from, hyperbilirubinemia in the period before total bilirubin reached 12 mg/dL. These factors included hepatic encephalopathy (HE), DIC, acute on chronic liver failure (ACLF), ischemic heart disease (IHD), stroke, organ dysfunction, cardiopulmonary resuscitation (CPR), extracorporeal membrane oxygenation (ECMO), liver surgery, and hypoxic hepatitis [4, 8]. Finally, we investigated the status of ursodeoxycholic acid (UDCA) medication during ICU stays to evaluate its effect on liver function improvement in extreme hyperbilirubinemia [19, 20].

HE was defined as altered consciousness as a result of liver failure due to liver disease excluding other causes for confusion or coma confirmed by liver function tests, ammonia measurement, liver US, brain CT, and electroencephalogram (EEG). DIC was defined as systemic activation of blood coagulation generating intravascular thrombosis blocking small blood vessels diagnosed using International Society on Thrombosis and Haemostasis (ISTH) scoring system [21]. ACLF was defined as the acute deterioration of liver function in patients with chronic liver disease determined using Asian-Pacific Association of the Liver (APASL) criteria [22]. IHD was defined as inadequate blood supply to the heart caused by narrowed coronary arteries such as angina or myocardial infarction confirmed by clinical symptoms and coronary angiography or stress testing such as exercise electrocardiogram (ECG), echocardiography, and myocardial perfusion scintigraphy. Stroke was defined as interrupted blood supply to the brain caused by blocked or burst blood vessels confirmed by neurological examination, brain CT, brain magnetic resonance imaging (MRI), and arteriography.

Organ dysfunction was defined as the SOFA score of 2 points or more. Hypoxic hepatitis was defined according to the following criteria: 1) clinical setting of cardiac, circulatory, or respiratory failure, 2) dramatic, but transient, increase in serum aminotransferase activity reaching at least 20-fold the upper limit of normal (normal ranges at the Samsung Medical Center, serum AST < 40 IU/L, serum ALT < 40 IU/L), and 3) exclusion of other potential causes of liver cell necrosis, particularly viral or drug-induced hepatitis and rhabdomyolysis [4, 12, 13, 23].

### Statistical analysis

The primary outcome variable was hospital mortality, defined as death occurring during hospital stay. The secondary outcome variable was recovery from hyperbilirubinemia, defined as decrease of the serum bilirubin level below 6 mg/dL which is SOFA score of 2 points or below regardless of whether the patients was in the ICU, general ward, or outpatient clinic after the peak serum bilirubin level.

We tested whether the peak serum bilirubin level and other clinical data were associated with hospital mortality and recovery from hyperbilirubinemia. The distributions of various factors were analyzed by independent T test, Chi-square test with Fisher’s exact test, Mann-Whitney test and Cochran-Armitage trend test. The differences in hospital mortality risk and the impact on recovery from extreme hyperbilirubinemia were tested using logistic regression analyses. All data were tested for normality and multivariable comparisons were adjusted by Bonferroni correction.

We used two models of stepwise multivariable logistic regression analyses with increasing degrees of adjustment to account for potential confounding and mediating factors. Model 1 was adjusted for age, sex, BMI, and medical history as follows: alcohol drinking, smoking, diabetes mellitus, hypertension, dyslipidemia, chronic liver disease, obstructive jaundice, cancer, hepatic encephalopathy, disseminated intravascular coagulation (DIC), acute on chronic liver failure (ACLF), ischemic heart disease, stroke, hypoxic hepatitis, sepsis, and shock. Model 2 was further adjusted for cardio-pulmonary resuscitation (CPR), extracorporeal membrane oxygenation (ECMO), liver surgery, ursodeoxycholic acid (UDCA), the initial Sequential Organ Failure Assessment (SOFA) score, number of dysfunctional organs, laboratory results, and type of hyperbilirubinemia.

The two most important causes of extreme hyperbilirubinemia in each patient were identified in order. These major factors’ associations with hospital mortality or bilirubin recovery were analyzed by categorized multiple response analysis with cross-tabulation and Chi-square test with Fisher’s exact test. Subsequently, the effects these factors had on extreme bilirubin increase were analyzed by ranked multiple response analysis. To ensure that the factors are weighted according to their level of importance, we multiplied the number of primary factors (N1) by 2, the number of secondary factors (N2) by 1, and the number of the other factors by 0. Then, we added them to obtain the influence index to evaluate each factor’s practical effect on bilirubin increase according to the importance [24–27].

All reported p values were two-sided and the significance level was set to 0.05. Statistical analyses were carried out with the Statistical Analysis System (SAS) version 9.4 (SAS Institute, Cary, NC).

## Results

### Baseline characteristics and comparison between survivors and non-survivors

This study included 427 patients with ages ranging from 19–98 years with a mean age of 59.8 ± 14.0 years. The number of males was 286 (67.0%) and females was 141 (33.0%). The mortality rate for the total population was 76.1% and the median duration of ICU stays was 12.3 (7.0~22.3) days. The proportion and mortality of medical ICU patients were higher than those of surgical ICU patients. The most common location before ICU admission was the ward accounting for around half of the total patients. The most common reason for ICU admission were respiratory followed by cardiovascular, which accounted for more than half of the total patients. Hematologic cancer, DIC, sepsis, and septic shock showed a higher occurrence in non-survivors than survivors. The mean score of initial SOFA and the mean number of dysfunctional organs were higher in non-survivors than survivors (Table 1). The median values of AST, INR, and lactate showed a higher level in non-survivors than survivors (Table 2).

**Table 1:** Baseline characteristics with comparison between survivors and non-survivors.

	All (N = 427)	Survivors (N = 102, 23.9%)	Non-survivors (N = 325, 76.1%)	p-value
Age (year, mean ± S.D.)	59.77 ± 13.97	58.35 ± 14.28	60.21 ± 13.86	0.242^a^
Sex				0.843^b^
Male	286 (67.0%)	67 (23.4%)	219 (76.6%)	
Female	141 (33.0%)	35 (24.8%)	106 (75.2%)	
BMI (kg/m^2^)	22.79±3.77	23.09±4.37	22.70±3.56	0.358^a^
Alcohol drinking	183 (42.9%)	45 (44.1%)	138 (42.5%)	0.768^b^
Smoking	172 (40.3%)	45 (44.1%)	127 (39.1%)	0.365^b^
Type of ICU				<0.001^b^
Medical	334 (78.2%)	66 (19.8%)	268 (80.2%)	
Surgical	93 (21.8%)	36 (38.7%)	57 (61.3%)	
Elective	37 (8.7%)	17 (45.9%)	20 (54.1%)	0.001^b^
Emergent	56 (13.1%)	19 (33.9%)	37 (66.1%)	0.059^b^
Location before ICU admission				<0.001^b^
ER	63 (14.8%)	18 (28.6%)	45 (71.4%)	0.345^b^
Ward	214 (50.1%)	32 (15.0%)	182 (85.0%)	<0.001^b^
OR	74 (17.3%)	31 (41.9%)	43 (58.1%)	<0.001^b^
OPD	5 (1.2%)	1 (20.0%)	4 (80.0%)	1.000^c^
Other hospital	71 (16.6%)	20 (28.2%)	51 (71.8%)	0.354^b^
Reason for ICU admission				<0.001^b^
Cardiovascular	94 (22.0%)	42 (44.7%)	52 (55.3%)	<0.001^b^
Respiratory	159 (37.2%)	25 (15.7%)	134 (84.3%)	0.002^b^
Gastrointestinal	65 (15.2%)	11 (16.9%)	54 (83.1%)	0.153^b^
Hepatobiliary-pancreatic	36 (8.4%)	12 (33.3%)	24 (66.7%)	0.165^b^
Urinary	25 (5.9%)	4 (16.0%)	21 (84.0%)	0.340^b^
Neurologic	14 (3.3%)	4 (28.6%)	10 (71.4%)	0.750^c^
Hematologic	5 (1.2%)	1 (20.0%)	4 (80.0%)	1.000^c^
Metabolic	8 (1.9%)	0 (0.0%)	8 (100.0%)	0.207^c^
Others	21 (4.9%)	3 (14.3%)	18 (85.7%)	0.290^b^
Comorbiditiy				
Diabetes mellitus	126 (29.5%)	27 (26.5%)	99 (30.5%)	0.441^b^
Hypertension	187 (43.8%)	48 (47.1%)	139 (42.8%)	0.446^b^
Dyslipidemia	26 (6.1%)	3 (2.9%)	23 (7.1%)	0.128^b^
Chronic liver disease	113 (26.5%)	23 (22.5%)	90 (27.7%)	0.304^b^
Cancer				
HBP cancer	98 (23.0%)	17 (16.7%)	81 (24.9%)	0.084^b^
Solid cancer (except HBP cancer)	103 (24.1%)	20 (19.6%)	83 (25.5%)	0.222^b^
Hematologic cancer	124 (29.0%)	18 (17.6%)	106 (32.6%)	0.004^b^
None	157 (36.8%)	55 (53.9%)	102 (31.4%)	<0.001^b^
Hepatic encephalopathy	35 (8.2%)	5 (4.9%)	30 (9.2%)	0.164^b^
DIC	255 (59.7%)	40 (39.2%)	215 (66.2%)	<0.001^b^
ACLF	94 (22.0%)	22 (21.6%)	72 (22.2%)	0.901^b^
Ischemic heart disease	20 (4.7%)	7 (6.9%)	13 (4.0%)	0.280^c^
Stroke	53 (12.4%)	11 (10.8%)	42 (12.9%)	0.568^b^
Acute kidney injury	365 (85.5%)	81 (79.4%)	284 (87.4%)	0.046^b^
Chronic kidney disease	77 (18.0%)	22 (21.6%)	55 (16.9%)	0.287^b^
Sepsis	254 (59.5%)	49 (48.0%)	205 (63.1%)	0.007^b^
Shock	269 (63.0%)	56 (54.9%)	213 (65.5%)	0.052^b^
Septic	187 (43.8%)	35 (34.3%)	152 (46.8%)	0.027^b^
Cardiogenic	32 (7.5%)	14 (13.7%)	18 (5.5%)	0.006^b^
Hypovolemic	51 (11.9%)	10 (9.8%)	41 (12.6%)	0.445^b^
CPR	64 (15.0%)	16 (15.7%)	48 (14.8%)	0.821^b^
ECMO	81 (19.0%)	26 (25.5%)	55 (16.9%)	0.054^b^
Liver surgery	21 (4.9%)	8 (7.8%)	13 (4.0%)	0.117^b^
Initial SOFA score	9.79 ± 2.37	9.29 ± 2.42	9.95 ± 2.33	0.014^a^
Number of dysfunctional organs	3.91 ± 1.14	3.56 ± 1.18	4.03 ± 1.11	<0.001^a^

SD: standard deviation; BMI: body mass index; ICU: intensive care unit; ER: emergency room; OR: operating room; OPD: out-patient department; HBP: hepatobiliary-pancreatic; DIC: disseminated intravascular coagulation; ACLF: acute on chronic liver failure; CPR: cardio-pulmonary resuscitation; ECMO: extracorporeal membrane oxygenation; SOFA: Sequential Organ Failure Assessment.

Non-survival: current hospital death.

Chronic liver disease: liver cirrhosis, chronic viral hepatitis, alcoholic liver disease, autoimmune hepatitis, drug-induced and toxic hepatitis.

Initial SOFA score: the score of SOFA at ICU admission.

^a^Independent T test

^b^Chi-square test

^c^Fisher’s exact test.

**Table 2:** Comparison of factors related to hyperbilirubinemia according to survival.

	All (N = 427)	Survivors (N = 102, 23.9%)	Non-survivors (N = 325, 76.1%)	p-value
Peak bilirubin value (mg/dL)	20.36 ± 10.87	19.38 ± 7.87	24.61 ± 11.37	<0.001^a^
Peak bilirubin grade				<0.001^e^
1 (12–15)	111 (26.0%)	42 (37.8%)	69 (62.2%)	
2 (15–20)	99 (23.2%)	28 (28.3%)	71 (71.7%)	
3 (20–30)	131(30.7%)	24 (18.3%)	107 (81.7%)	
4 (30-)	86(20.1%)	8 (9.3%)	78 (90.7%)	
Laboratory analyses (median [IQR])				
AST (IU/L)	119.00 (53.60~467.43)	83.86 (47.43~210.27)	135.69 (58.90~594.25)	0.013^b^
ALT (IU/L)	76.00 (36.65~235.74)	56.97 (32.78~188.81)	82.19 (37.92~247.07)	0.127^b^
ALP (IU/L)	121.50 (83.29~200.25)	115.49 (80.64~180.00)	122.85 (84.46~201.00)	0.400^b^
GGT (IU/L)	79.27 (41.70~185.54)	87.33 (47.67~209.00)	77.29 (40.80~180.75)	0.317^b^
INR	1.65 (1.40~2.08)	1.47 (1.24~1.75)	1.71 (1.47~2.16)	<0.001^b^
Albumin (g/dL)	2.78 (2.53~3.03)	2.83 (2.61~3.01)	2.77 (2.51~3.06)	0.134^b^
CRP (mg/dL)	10.21 (5.50~15.42)	9.80 (5.58~14.04)	10.35 (5.48~15.68)	0.291^b^
Lactate (mg/dL)	3.44 (2.32~5.36)	2.33 (1.73~3.44)	3.87 (2.71~6.35)	<0.001^b^
Cause of hyperbilirubinemia^f^				
Obstructive	40	16	24	
Stone	14 (3.3%)	7 (6.9%)	7 (2.2%)	0.048^d^
Stricture	23 (5.4%)	8 (7.8%)	15 (4.6%)	0.208^c^
Neoplasm	3 (0.7%)	1 (1.0%)	2 (0.6%)	0.560^d^
Non-obstructive	814	188	626	
Hypoxic hepatitis	141 (33.0%)	25 (24.5%)	116 (35.7%)	0.036^c^
Infection including sepsis	270 (63.2%)	62 (60.8%)	208 (64.0%)	0.557^c^
Primary liver disease	115 (26.9%)	21 (20.6%)	94 (28.9%)	0.098^c^
Traumatic or surgical liver injury	14 (3.3%)	5 (4.9%)	9 (2.8%)	0.338^d^
Pigment overload	90 (21.1%)	27 (26.5%)	63 (19.4%)	0.126^c^
Drug	107 (25.1%)	33 (32.4%)	74 (22.8%)	0.051^c^
Other organ failure-relation	71 (16.6%)	15 (14.7%)	56 (17.2%)	0.550^c^
Long-term NPO	6 (1.4%)	0 (0.0%)	6 (1.8%)	0.343^d^
Type of hyperbilirubinemia				0.125^c^
Direct	341 (84.8%)	87 (25.5%)	254 (74.5%)	
Indirect	61 (15.2%)	10 (16.4%)	51 (83.6%)	

IQR: interquartile range; IU: international unit; AST: aspartate aminotransferase; ALT: alanine aminotransferase; ALP: alkaline phosphatase; GGT: γ(gamma)-glutamyl transferase; INR: international normalized ratio; CRP: c-reactive protein; NPO: nil-per-os.

Non-survival: current hospital death.

Laboratory analyses: median values of calculated means in each and every patient obtained during the 15 days before and after the first incidence of serum total bilirubin ≧ 12 mg/dL after ICU admission

Cause of hyperbilirubinemia: the factor identified as two most important causes of extreme hyperbilirubinemia in each patient

^a^Independent T test

^b^Mann-Whitney test

^c^Chi-square test

^d^Fisher’s exact test

^e^Cochran-Armitage trend test

^f^Categorized multiple response analysis.

**Fig 1:**
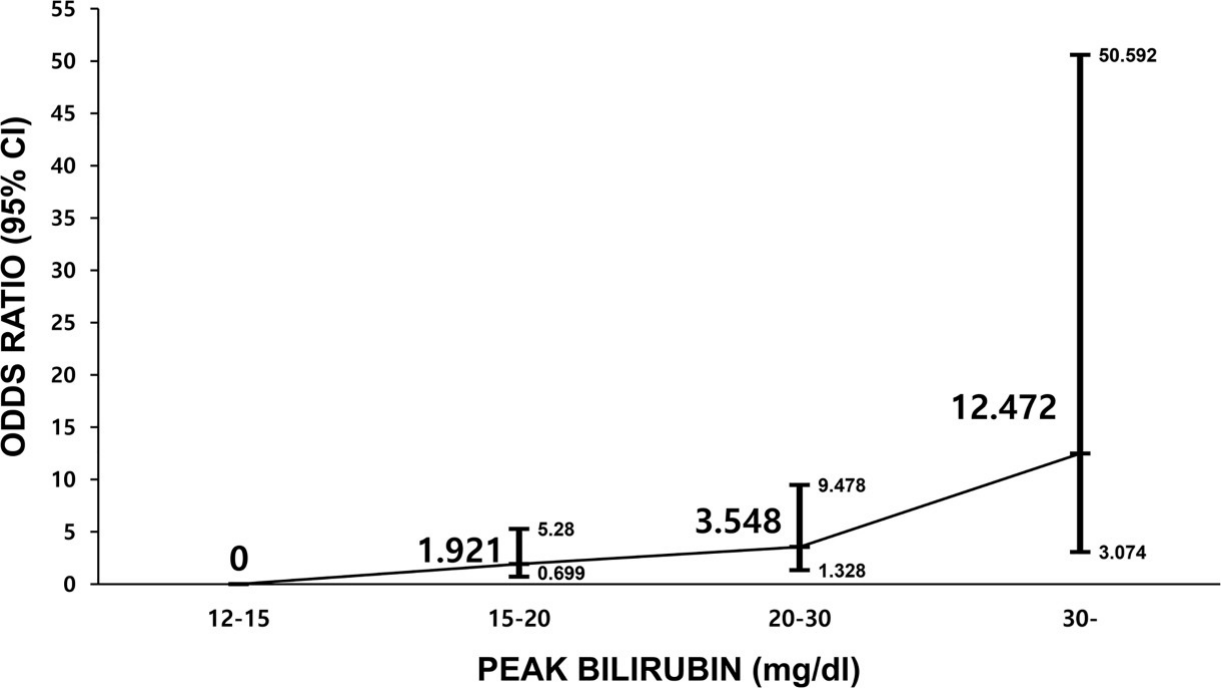
Relative mortality risk according to the peak bilirubin grade (multivariable logistic analysis). Model 2, multivariable logistic analysis with sex, age, body mass index (BMI), medical history (alcohol drinking, smoking, diabetes mellitus, hypertension, dyslipidemia, chronic liver disease, obstructive jaundice, cancer, hepatic encephalopathy, disseminated intravascular coagulation (DIC), acute on chronic liver failure (ACLF), ischemic heart disease, stroke, hypoxic hepatitis, sepsis, and shock), cardio-pulmonary resuscitation (CPR), extracorporeal membrane oxygenation (ECMO), liver surgery, ursodeoxycholic acid (UDCA), the initial Sequential Organ Failure Assessment (SOFA) score, number of dysfunctional organs, laboratory results, and type of hyperbilirubinemia. Abbreviation: OR, odds ratio; CI, confidence interval.

The mean value of peak bilirubin was 20.36 ± 10.9 mg/dL and was higher in non-survivors than survivors (24.6 mg/dL vs. 19.4 mg/dL, p < 0.001). The survival rate was significantly decreased as extreme hyperbilirubinemia grades increased; 37.8%, 28.3%, 18.3%, and 9.3% respectively for grades 1–4 (p < 0.001) (Table 2). The peak bilirubin value (per mg/dL) was significantly related to hospital mortality in model 2 analysis (OR, 1.09; 95% CI, 1.04–1.15, p < 0.001). Compared to that of grade 1 extreme hyperbilirubinemia, the risk of mortality gradually increased as the grade increased in model 2 analysis (OR [95% CI]: 1.92 [0.70–5.28], 3.55 [1.33–9.48], and 12.47 [3.07–50.59] for grades 2–4, respectively) (Table 3, Fig 1). In addition, we identified that the mortality risk in each hyperbilirubinemia grade gradually increased from crude analysis to model 2 analysis as more confounding variables were adjusted (Table 3).

**Table 3:** Odds ratios for mortality according to the peak bilirubin level.

		N	Hospital mortality (%)	Crude OR (95% CI)	Model 1 OR (95% CI)	Model 2 OR (95% CI)
Peak bilirubin value (mg/dL)				1.062 (1.025–1.099)	1.075 (1.029–1.124)	1.093 (1.042–1.147)
Peak bilirubin grade						
1	(12~15)	111	62.2	Reference	Reference	Reference
2	(15~20)	99	71.7	1.543 (0.759–3.141)	1.657 (0.734–3.742)	1.921 (0.699–5.280)
3	(20~30)	131	81.7	2.714 (1.327–5.549)	3.350 (1.493–7.520)	3.548 (1.328–9.478)
4	(30~)	86	90.7	5.935 (2.174–16.203)	8.315 (2.761–25.035)	12.472 (3.074–50.592)

OR: odds ratio; CI, confidence interval.

Chronic liver disease: liver cirrhosis, chronic viral hepatitis, alcoholic liver disease, autoimmune hepatitis, drug-induced and toxic hepatitis.

Model 1, multivariable logistic analysis with sex, age, body mass index (BMI), medical history (alcohol drinking, smoking, diabetes mellitus, hypertension, dyslipidemia, chronic liver disease, obstructive jaundice, cancer, hepatic encephalopathy, disseminated intravascular coagulation (DIC), acute on chronic liver failure (ACLF), ischemic heart disease, stroke, hypoxic hepatitis, sepsis, and shock); Model 2, multivariable logistic analysis with sex, age, BMI, medical history (same as model 1), cardio-pulmonary resuscitation (CPR), extracorporeal membrane oxygenation (ECMO), liver surgery, ursodeoxycholic acid (UDCA), the initial Sequential Organ Failure Assessment (SOFA) score, number of dysfunctional organs, laboratory results, and type of hyperbilirubinemia.

**Fig 2:**
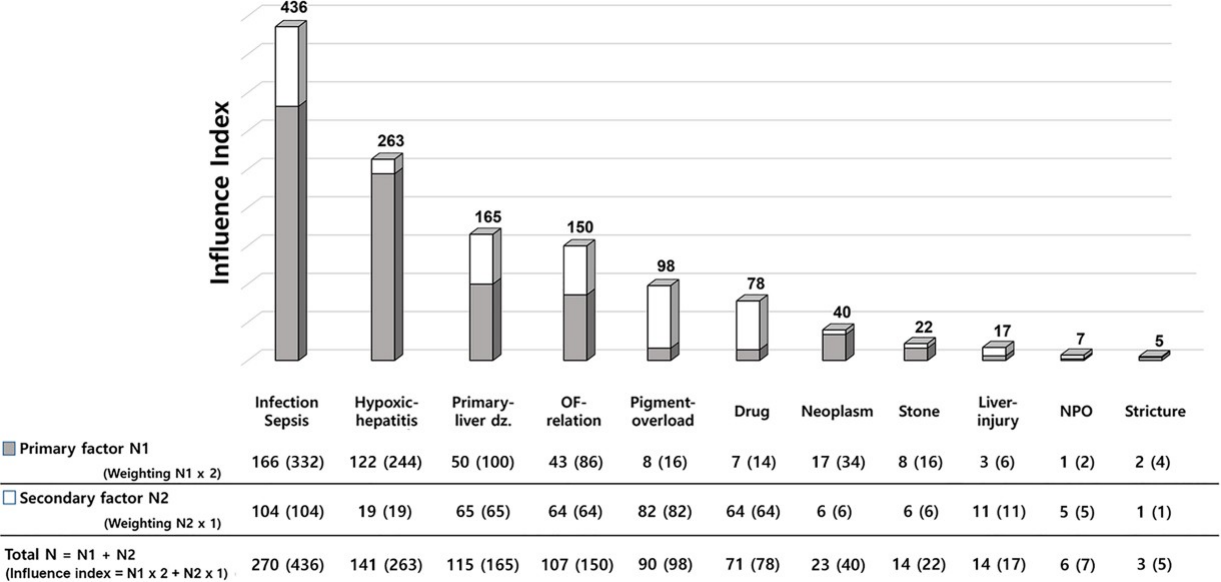
Influence index of main factors causing extreme hyperbilirubinemia (ranked multiple response analysis). Primary factor: the 1^st^ important cause of extreme hyperbilirubinemia in each patient. N1: the number of factors identified as Primary factor. Secondary factor: the 2^nd^ important cause of extreme hyperbilirubinemia in each patient. N2: the number of factors identified as Secondary factor. Abbreviation: dz., disease; OF, organ failure; NPO, nil-per-os.

Regarding the cause of hyperbilirubinemia, a total of 46 patients with obstructive jaundice were confirmed. In categorized multiple response analysis, obstructive jaundice was the major cause of extreme hyperbilirubinemia in 40 cases, whereas non-obstructive jaundice was the major cause of extreme hyperbilirubinemia in 814 cases. Among the cases, stone showed a higher rate in survivors, whereas hypoxic hepatitis showed a lower rate in survivors than non-survivors. The proportion of hospital mortality was higher in the cases suffering from non-obstructive jaundice than obstructive jaundice (626/814 [76.9%] vs. 24/40 [60.0%]) (Table 2). As described in the Methods section, the influence of the major causes of extreme hyperbilirubinemia was quantified by weighting and summing with ranked multiple response analysis, which were ranked in the following order according to the influence score: infection including sepsis (436), hypoxic hepatitis (263), primary liver disease (165), other organ failure-related jaundice (150), pigment overload (98), drug (78), neoplasm (40), stone (22), liver injury (17), NPO (7), and stricture (5) (Fig 2).

Regarding the type of hyperbilirubinemia, total 402 patients were assessed (excluding 25 patients who did not have serum direct bilirubin levels measured). The number of patients with direct hyperbilirubinemia (DB / TB ≧ 60%) was 341 (84.8%) and indirect hyperbilirubinemia was 61 (15.2%). Direct hyperbilirubinemia showed a higher rate in survivors, whereas indirect hyperbilirubinemia showed a lower rate in survivors than in non-survivors. The serum direct bilirubin levels of 44 patients among the 46 patients with obstructive jaundice were measured, and they were examined for direct hyperbilirubinemia. Most patients with obstructive jaundice (40/44, 90.9%) had direct hyperbilirubinemia, and the proportion of obstructive jaundice was higher in the direct than the indirect hyperbilirubinemia patients (40/341 [11.7%] vs. 4/61 [6.6%]) (Table 2).

### Comparison between recovery and non-recovery group from extreme hyperbilirubinemia

Recovery of hyperbilirubinemia to SOFA grades (points) 2 or below was observed in 110 (25.8%) patients. From Table 4, we did not identify any difference in age, sex, and history of alcohol drinking and smoking between the group that recovered and the group that did not recover from extreme hyperbilirubinemia. Chronic liver disease, cancer, HE, DIC, ACLF, and shock appeared at a higher occurrence in the non-recovery than the recovery group. The mean score of initial SOFA and the mean number of dysfunctional organs were higher in the non-recovery than the recovery group. UDCA was administered at a higher rate in the recovery than the non-recovery group. The median AST, INR, and lactate showed a higher level in the non-recovery than the recovery group (Table 4).

**Table 4:** Comparison of patient characteristics between recovery and non-recovery groups.

	Recovery Group (N = 110, 25.8%)	Non-recovery Group (N = 317, 74.2%)	p-value
Age (year, mean ± S.D.)	58.20 ± 14.40	60.31 ± 13.79	0.173^a^
Sex			0.455^c^
Male	70 (24.5%)	216 (75.5%)	
Female	40 (28.4%)	101 (71.6%)	
Alcohol drinking	44 (40.0%)	139 (43.8%)	0.482^c^
Smoking	40 (36.4%)	132 (41.6%)	0.331^c^
Comorbiditiy			
Diabetes mellitus	30 (27.3%)	96 (30.3%)	0.551^c^
Hypertension	53 (48.2%)	134 (42.3%)	0.282^c^
Dyslipidemia	5 (4.5%)	21 (6.6%)	0.432^c^
Chronic liver disease	16 (14.5%)	97 (30.6%)	0.001^c^
Cancer			0.004^c^
Yes	57 (51.8%)	213 (67.2%)	
None	53 (48.2%)	104 (32.8%)	
Hepatic encephalopathy	4 (3.6%)	31 (9.8%)	0.043^c^
DIC	49 (44.5%)	206 (65.0%)	<0.001^c^
ACLF	14 (12.7%)	80 (25.2%)	0.006^c^
Acute kidney injury	86 (78.2%)	279 (88.0%)	0.012^c^
Chronic kidney disease	23 (20.9%)	54 (17.0%)	0.362^c^
Sepsis	58 (52.7%)	196 (61.8%)	0.094^c^
Shock	58 (52.7%)	211 (66.6%)	0.010^c^
Septic	43 (39.1%)	144 (45.4%)	0.249^c^
Cardiogenic	11 (10.0%)	21 (6.6%)	0.247^c^
Hypovolemic	8 (7.3%)	43 (13.6%)	0.080^c^
CPR	19 (17.3%)	45 (14.2%)	0.436^c^
ECMO	26 (25.5%)	55 (16.9%)	0.054^c^
Liver surgery	7 (6.4%)	14 (4.4%)	0.416^c^
UDCA administration	35 (31.8%)	62 (19.6%)	0.008^c^
Initial SOFA score	9.34 ± 2.52	9.95 ± 2.29	0.018^a^
Number of dysfunctional organs	3.68 ± 1.27	3.99 ± 1.09	0.023^a^
Peak bilirubin value (mg/dL)	18.10 ± 6.71	25.19 ± 11.43	<0.001^a^
Peak bilirubin grade			<0.001^e^
1 (12–15)	52 (46.8%)	59 (53.2%)	
2 (15–20)	29 (29.3%)	70 (70.7%)	
3 (20–30)	24 (18.3%)	107 (81.7%)	
4 (30-)	5 (5.8%)	81 (94.2%)	
Laboratory analyses (median (IQR))			
AST (IU/L)	84.27 (42.18~207.25)	136.09 (59.53~617.88)	<0.001^b^
ALT (IU/L)	59.96 (29.46~193.44)	82.19 (38.54~247.50)	0.073^b^
ALP (IU/L)	108.71 (78.92~163.33)	129.00 (85.13~203.10)	0.052^b^
GGT (IU/L)	87.67 (53.07~205.00)	76.17 (40.00~182.00)	0.121^b^
INR	1.43 (1.24~1.63)	1.75 (1.51~2.25)	<0.001^b^
Albumin (g/dL)	2.85 (2.59~3.03)	2.77 (2.51~3.03)	0.076^b^
CRP (mg/dL)	11.23 (5.76~15.27)	9.60 (5.34~15.45)	0.402^b^
Lactate (mg/dL)	2.46 (1.76~3.71)	3.81 (2.70~6.53)	<0.001^b^
Cause of hyperbilirubinemia^f^			
Obstructive	17	23	
Stone	6 (5.5%)	8 (2.5%)	0.209^d^
Stricture	9 (8.2%)	14 (4.4%)	0.132^c^
Neoplasm	2 (1.8%)	1 (0.3%)	0.164^d^
Non-obstructive	203	611	
Hypoxic hepatitis	28 (25.5%)	113 (35.6%)	0.050^c^
Infection including sepsis	70 (63.6%)	200 (63.1%)	0.919^c^
Primary liver disease	12 (10.9%)	103 (32.5%)	<0.001^c^
Traumatic or surgical liver injury	4 (3.6%)	10 (3.2%)	0.762^d^
Pigment overload	27 (24.5%)	63 (19.9%)	0.301^c^
Drug	39 (35.5%)	68 (21.5%)	0.003^c^
Other organ failure-relation	22 (20.0%)	49 (15.5%)	0.270^c^
Long-term NPO	1 (0.9%)	5 (1.6%)	1.000^d^
Type of hyperbilirubinemia			0.005^c^
Direct	98 (28.7%)	243 (71.3%)	
Indirect	7 (11.5%)	54 (88.5%)	
Hospital mortality	32 (29.1%)	293 (92.4%)	<0.001^c^

SD: standard deviation; DIC: disseminated intravascular coagulation; ACLF: acute on chronic liver failure; CPR: cardio-pulmonary resuscitation; ECMO: extracorporeal membrane oxygenation; UDCA: ursodeoxycholic acid, SOFA, Sequential Organ Failure Assessment; IQR: interquartile range; IU: international unit; AST: aspartate aminotransferase; ALT: alanine aminotransferase; ALP: alkaline phosphatase; GGT: γ(gamma)-glutamyl transferase; INR: international normalized ratio; CRP: c-reactive protein; NPO: nil-per-os.

Recovery group: the patients whose serum bilirubin decreased below 6 mg/dL.

Non-recovery group: the patients whose serum bilirubin didn’t decrease below 6 mg/dL.

Chronic liver disease: liver cirrhosis, chronic viral hepatitis, alcoholic liver disease, autoimmune hepatitis, drug-induced and toxic hepatitis.

Initial SOFA score: the score of SOFA at ICU admission.

Laboratory analyses: median values of calculated means in each and every patient obtained during the 15 days before and after the first incidence of serum total bilirubin ≧ 12 mg/dL after ICU admission.

Cause of hyperbilirubinemia: the factor identified as two most important causes of extreme hyperbilirubinemia in each patient.

^a^Independent T test

^b^Mann-Whitney test

^c^Chi-square test

^d^Fisher’s exact test

^e^Cochran-Armitage trend test

^f^Categorized multiple response analysis.

**Fig 3:**
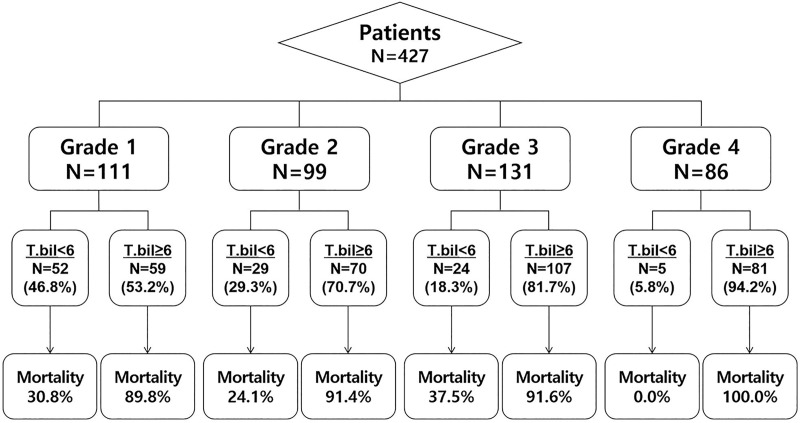
Bilirubin recovery (< 6 mg/dL) or non-recovery from extreme hyperbilirubinemia and subsequent mortality according to the peak bilirubin grade. Abbreviation: Bilirubin recovery, bilirubin decrease below 6 mg/dL.

The mean value of peak bilirubin was lower in the recovery than the non-recovery group (18.10 mg/dL vs. 25.19 mg/dL, p < 0.001). The recovery rate significantly decreased as extreme hyperbilirubinemia grades increased. Mortality for all patients and mortality associated with each grade of extreme hyperbilirubinemia were lower in recovery group than in their non-recovery group counterparts (overall mortality, 29.1% vs. 92.4%, p < 0.001) (Table 4, Fig 3).

Regarding the cause of hyperbilirubinemia, primary liver disease showed a lower rate in the recovery group, whereas the usage of drug showed a higher rate in the recovery than the non-recovery group. The proportion of bilirubin recovery was higher in the cases suffering from obstructive jaundice than non-obstructive jaundice (17/40 [42.5%] vs. 203/814 [24.9%]) (Table 4).

Regarding the type of hyperbilirubinemia, direct hyperbilirubinemia showed a higher rate in the recovery group, whereas indirect hyperbilirubinemia showed a lower rate in the recovery than the non-recovery group (Table 4).

**Fig 4:**
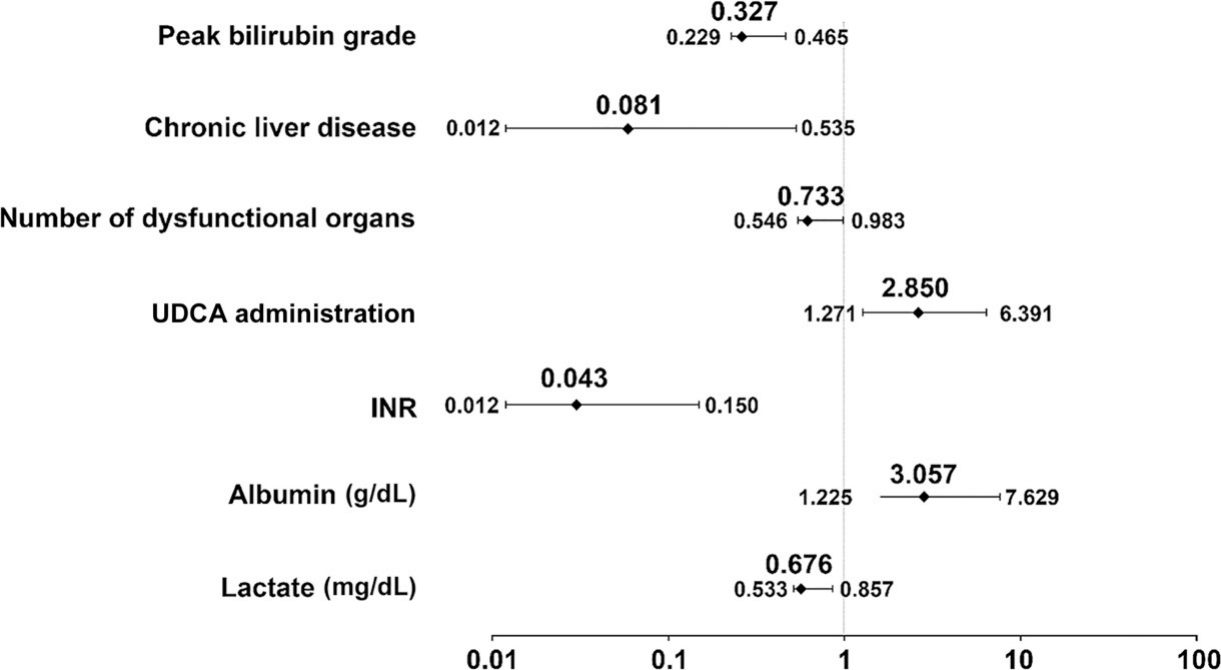
Relative impact of variables on bilirubin recovery (multivariable logistic analysis described with OR and 95% CI). Model 2, multivariable logistic analysis with sex, age, body mass index (BMI), medical history (alcohol drinking, smoking, diabetes mellitus, hypertension, dyslipidemia, chronic liver disease, obstructive jaundice, cancer, hepatic encephalopathy, disseminated intravascular coagulation (DIC), acute on chronic liver failure (ACLF), ischemic heart disease, stroke, hypoxic hepatitis, sepsis, and shock), cardio-pulmonary resuscitation (CPR), extracorporeal membrane oxygenation (ECMO), liver surgery, ursodeoxycholic acid (UDCA), the initial Sequential Organ Failure Assessment (SOFA) score, number of dysfunctional organs, laboratory results, and type of hyperbilirubinemia. Abbreviation: Bilirubin recovery, bilirubin decrease below 6 mg/dL; OR, odds ratio; CI, confidence interval; UDCA, ursodeoxycholic acid; INR, international normalized ratio.

UDCA and albumin were identified as favorable factors for recovery from extreme hyperbilirubinemia, whereas peak bilirubin grade, chronic liver disease, the number of dysfunctional organs, INR, and lactate were unfavorable factors (Fig 4).

## Discussion

The purposes of this study were to assess the usefulness of the subdividing extreme hyperbilirubinemia when determining the prognosis of critically ill patients and to evaluate the clinical course of disease in patients with extreme hyperbilirubinemia. The study results demonstrate a clear difference in patients’ clinical outcomes based on novel subcategorized level of extreme hyperbilirubinemia. Multivariable analysis using the logistic regression model showed a significant trend of increasing hospital mortality with increased hyperbilirubinemia grade. Furthermore, recovery from hyperbilirubinemia was inversely related to the grade of hyperbilirubinemia and hospital mortality. These results support that both extreme hyperbilirubinemia and recovery from extreme hyperbilirubinemia could be important prognostic factors that clinicians should follow to predict the clinical course of critically ill patients.

A recent study reported that hepatic dysfunction, which is defined as a total bilirubin increase above 2 mg/dL, occurs in 31% of critically ill patients and shows independent roles in clinical outcomes [5]. Furthermore, the risk of death in critically ill patients was more closely related to hepatic dysfunction than to other prognostic values [1–5, 28]. In patients with hepatic dysfunction, canalicular bile secretion is reduced [29], and reduced biliary secretion could be considered the main component of hepatic dysfunction in critically ill patients [30]. Thus, although several previous studies have suggested other markers to assess hepatic dysfunction (HE, ascites, or elevated serum aspartate aminotransferase or alkaline phosphatase), serum bilirubin is currently considered a stable and powerful marker of hepatic dysfunction [6, 7, 31–33].

Most commonly, serum total bilirubin levels greater than 2 mg/dL are used as a threshold for hepatic dysfunction. Many studies have analyzed the prognostic value of hyperbilirubinemia above 2 mg/dL; however, they only analyzed total bilirubin below 12 mg/dL [3, 5, 34]. As mentioned above, extreme hyperbilirubinemia could be an important prognostic factor in critically ill patients, but no predictive model has been established to further grade total bilirubin above 12 mg/dL. Thus, assessing the prognostic value of total bilirubin above 12 mg/dL is essential. In addition, our study determined that recovery from hyperbilirubinemia could be also an important prognostic factor in patients with extreme hyperbilirubinemia. Furthermore, given the association between various factors and highly elevated bilirubin during the clinical course based on a review of the medical records and the opinions of experts, we found that various factors collectively contribute to the development of extreme hyperbilirubinemia.

Various factors affecting hyperbilirubinemia have been studied [4, 8, 10, 11]; however, it has been difficult to specify how much these factors affect bilirubin increase and what their order of importance is. By analyzing patients’ clinical features along with the progress of extreme hyperbilirubinemia, we could quantify the factors’ influence. Regarding the order of importance, our study verified that an infection including sepsis, hypoxic hepatitis, primary liver disease, and other organ failure-related jaundice could be main causes of extreme hyperbilirubinemia. Furthermore, we confirmed that various factors work together simultaneously to lead to the development of extreme hyperbilirubinemia.

Our study showed that the high albumin concentration and use of UDCA were correlated with recovery from extreme hyperbilirubinemia. UDCA has been previously recognized to be effective in improving liver function [19, 20], and we identified that UDCA exerted a positive effect on bilirubin recovery from extreme hyperbilirubinemia as well. Albumin administration appeared to improve renal failure in liver dysfunction [35], which can contribute to preventing the deterioration of liver dysfunction and lead to recovery. Meanwhile, consistent with previous studies of hyperbilirubinemia [10, 36], we verified that underlying liver disease, hepatic dysfunction, and metabolic acidosis or hypo-perfusion state (sepsis and shock) could impede bilirubin recovery from extreme hyperbilirubinemia.

This study is significant because it is the first large-scale cohort study that shows the correlation between extreme hyperbilirubinemia and the mortality or recovery of critically ill patients. In addition, it is an unprecedented study that determines what factors cause extreme hyperbilirubinemia while presenting these factors’ influences as specified index in figures. The findings of this study can be applied to extreme hyperbilirubinemia patients to predict their prognosis and concurrently provide baseline data for future investigations focusing on predicting the clinical courses of patients with extremely high bilirubin.

However, we did not analyze the statistical cut-off value for peak bilirubin levels related to mortality and recovery in this study. Instead, we simply divided patients into four extreme hyperbilirubinemia grades with the arithmetic number of peak bilirubin levels: these are easier to apply in clinical practices. There was an obvious gap between the OR between grade 3 and 4; however, there could be another cut-off value related to mortality for total bilirubin over 30 mg/dL. Therefore, further analysis about the statistical cut-off value of extreme hyperbilirubinemia needs to be conducted.

This study has some limitations. First, this is retrospective study and therefore may include natural biases such as selection bias and reporting bias. Second, there could be other pre-existing medical conditions related to hyperbilirubinemia and recovery that were not included. Thus, it is possible that hidden prognostic factors were not evaluated in this study. Critically ill patients are a heterogeneous population, so it is difficult to say whether the heterogeneity of the clinical features does not have any influence. Last, despite the close and detailed examination, selecting the causes of extreme hyperbilirubinemia was somewhat subjective and report. As a result, further large-scale prospective studies with a more objective validation are required.

## Conclusion

In conclusion, the subdivided extreme hyperbilirubinemia levels (total bilirubin ≥ 12 mg/dL) have valuable association with mortality and recovery for critically ill patients. This study shows that it is important to assess the level of extreme hyperbilirubinemia during ICU stay for predicting prognosis for critically ill patients. Further statistical analyses and a well-designed prospective validation study for the classification of extreme hyperbilirubinemia in critically ill patients are necessary.

## Supporting information

S1 Fig The selection process of the study population for extreme hyperbilirubinemia. (TIF)

S1 Dataset (XLSX)

## Abbreviations

ACLF: acute on chronic liver failure
ALP: alkaline phosphatase
ALT: alanine aminotransferase
APASL: Asian-pacific association of the liver
AST: aspartate aminotransferase
BMI: body mass index
CI: confidence interval
CPR: cardio-pulmonary resuscitation
CRP: c-reactive protein
CT: computerized-tomography
DB: direct bilirubin
DIC: disseminated intravascular coagulation
DM: diabetes mellitus
ECG: electrocardiogram
ECMO: extracorporeal membrane oxygenation
GCS: Glasgow Coma Scale
GGT: γ(gamma)-glutamyl transferase
HBP: hepatobiliary-pancreatic
HE: hepatic encephalopathy
HTN: hypertension
ICU: intensive care unit
IHD: ischemic heart disease
INR: international normalized ratio
IRB: Institutional Review Board
ISTH: international society on thrombosis and haemostasis
IU: international unit
MRI: magnetic resonance imaging
NPO: null-per-os
OR: odds ratio
SAS: Statistical Analysis System
SOFA: sequential organ failure assessment
TB: total bilirubin
TPN: total parenteral nutrition
UDCA: ursodeoxycholic acid
US: ultra-sonography
