# Supplementary figures and images for: Evaluating mortality and recovery of extreme hyperbilirubinemia in critically ill patients by phasing the peak bilirubin level: A retrospective cohort study
Source: PLoS One. 2021 Aug 5;16(8):e0255230. doi: 10.1371/journal.pone.0255230 (PMC8341602; doi:10.1371/journal.pone.0255230)

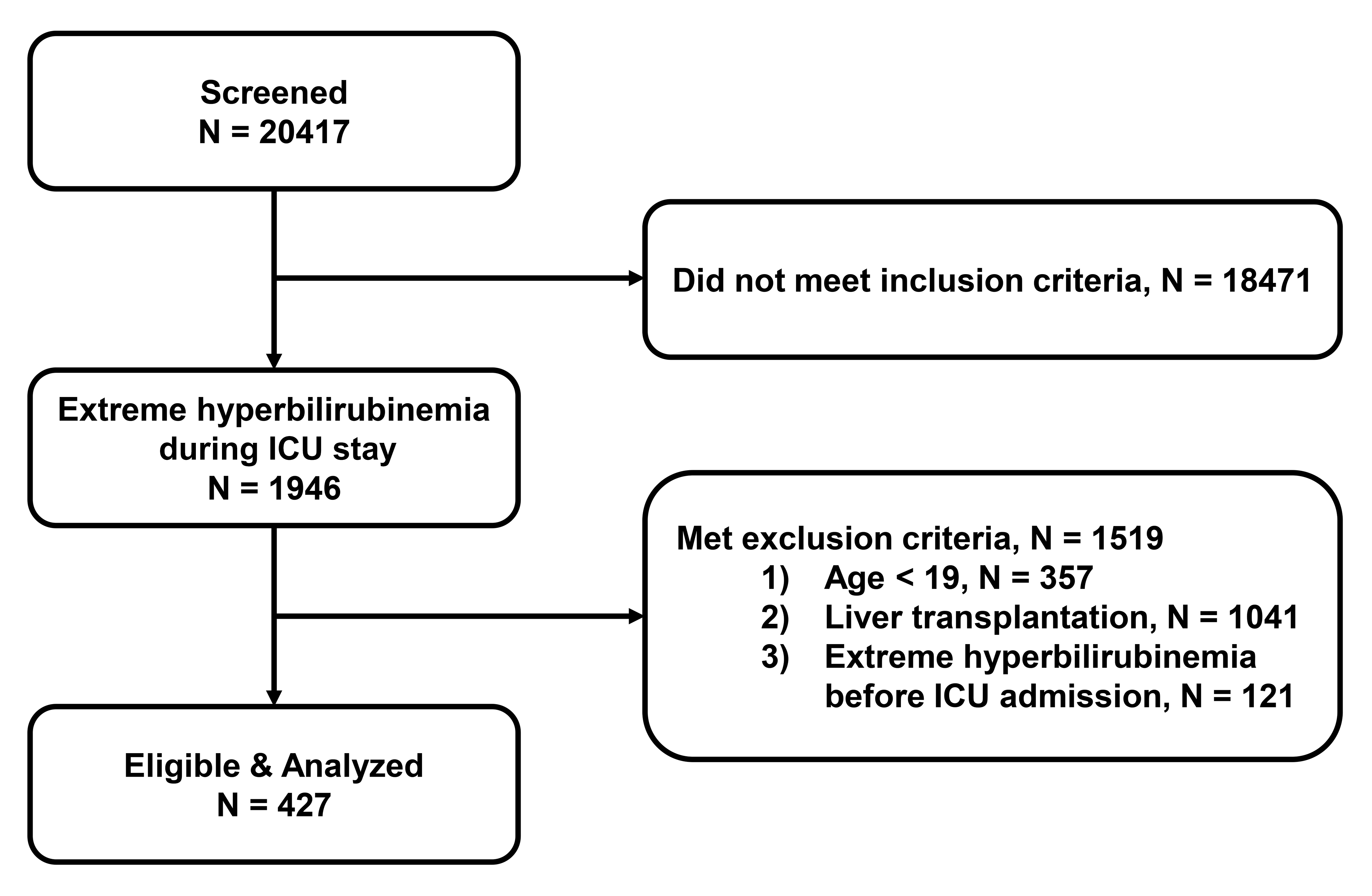

Supplement: S1 Fig — (TIF) [file pone.0255230.s001.tif]
